# Coronary sinus and cardiac venous anatomy for cardiac resynchronization therapy: A Clinician's view

**DOI:** 10.1016/j.ipej.2026.06.003

**Published:** 2026-06-12

**Authors:** Maxim Didenko, Karen Harutyunyan, Christoph Scharf, Moneeb Khalaph, Thomas Fink, Thomas Eitz, Ersan Akkaya, Guram Imnadze, Yuri Bocchini, Hassan El-Shirbiny, Martin Braun, Angeliki Darma, Vanessa Sciacca, Christian Sohns, Philipp Sommer

**Affiliations:** a Department of Electrophysiology, Heart and Diabetes Center NRW, Ruhr University Bochum, Bad Oeynhausen, Germany; b Rhythmologie Zürich AG, Zürich, Switzerland; c Clinic for Electrophysiology, University Hospital Ruppin-Brandenburg, Germany; d Cardiology Department, Faculty of Medicine, Kafr Elsheikh University, Kafr El Sheikh, Egypt

**Keywords:** Cardiac resynchronization therapy, Coronary sinus anatomy, Left ventricular lead, Coronary venous system, Cardiac imaging, Heart failure

## Abstract

The coronary sinus and its tributaries constitute the anatomical foundation for successful cardiac resynchronization therapy. Despite the emergence of conduction system pacing as a physiological alternative, conventional transvenous CRT retains its important role—not all clinical scenarios can be addressed by left bundle branch area or His bundle pacing alone. Understanding coronary venous anatomy therefore remains essential for every implanting physician. This review provides a clinically oriented analysis of coronary venous anatomy as it pertains to left ventricular lead implantation.

We examine the embryological origins of the coronary sinus, explaining why variants such as persistent left superior vena cava and obstructive Thebesian and Vieussens valves occur. The gross anatomy section details coronary sinus dimensions, ostial localization, and tributary classification using attitudinally correct nomenclature. Microanatomical considerations include wall thickness gradients, subepicardial adipose tissue thickness, and myocardial sleeve distribution.

We review imaging modalities—fluoroscopy, computed tomography, and magnetic resonance imaging—emphasizing their complementary roles in preprocedural planning and real-time guidance. Catheter and wire handling techniques are discussed, from cannulation strategies to lead delivery. Electrophysiological parameters including Q-LV and S-QRS intervals are examined in the context of anatomical lead positioning.

Finally, we analyze how anatomy influences outcomes, complications, and non-response. Understanding the coronary venous system transforms cardiac resynchronization therapy from a technical procedure into an anatomically informed intervention where success depends on matching therapeutic goals with individual patient anatomy.

## 1 Introduction

If you've ever struggled to cannulate a coronary sinus (CS) ostium hidden behind a thick Thebesian valve, or watched helplessly as a perfectly positioned lead migrated out of a straight lateral vein during the first post-op cough, you already know that successful cardiac resynchronization therapy (CRT) isn't just about following guidelines. It's about understanding anatomy. The coronary venous system, elegant in its complexity and frustrating in its variability, determines whether our best intentions translate into improved ejection fractions and reduced heart failure hospitalizations.

Why does CS anatomy remain critically relevant in the era of conduction system pacing? Because we have learned that not all problems can be solved with CSP. Left bundle branch area pacing and His bundle pacing offer physiological alternatives, but anatomical challenges, insufficient capture, and specific patient populations ensure that conventional transvenous CRT will retain its important role. Understanding the coronary venous system remains essential for every implanting physician.

This review takes you through the CS from its embryological origins to the technical nuances of lead placement, with one goal in mind: helping you anticipate anatomical challenges before they become procedural failures. We'll examine not just what the anatomy looks like, but why it matters when you're standing in the lab with limited venous options and a patient who really needs this therapy to work.

Throughout this review, we use attitudinally appropriate anatomical nomenclature as recommended by recent consensus statements [1]. The diaphragmatic surface of the heart is positioned inferiorly, not posteriorly, relative to the body coordinates. Accordingly, structures traditionally described as "posterior" (e.g., posterior cardiac vein, posterolateral vein) are more accurately termed "inferior" or "inferolateral." This approach aligns anatomical description with fluoroscopic and computed tomography (CT) imaging perspectives, where the inferior border of the heart clearly sits on the diaphragm.

**Fig. 1:**
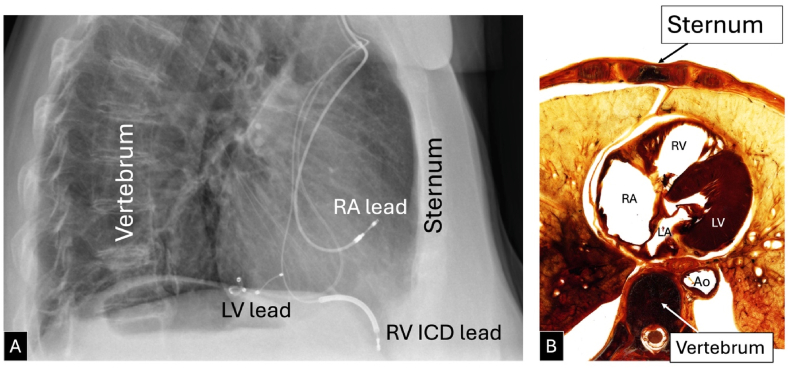
Spatial relationship between right and left ventricular leads: the left ventricle is positioned posteriorly. (A) Lateral chest radiograph following CRT-D implantation. The left ventricular lead, positioned in a lateral coronary vein, courses posteriorly relative to the right ventricular ICD lead. This projection demonstrates that "lateral" veins of the left ventricle are actually positioned posteriorly within the body - a spatial relationship essential for interpreting fluoroscopic projections during implantation. The sternum and vertebral column provide anatomical reference points. (B) Transverse section of a human cadaveric thorax at the mid-ventricular level, corresponding to a standard axial CT plane. This anatomical cross-section confirms the posterior position of the left ventricle relative to the right ventricle, explaining why catheter manipulation toward the lateral left ventricular wall requires posterior-directed movement in the body coordinate system. Abbreviations: Ao, aorta; LA, left atrium; LV, left ventricle; RA, right atrium; RV ICD lead, right ventricle defibrillation lead.

Fig. 1 illustrates this spatial relationship: the left ventricle is positioned posteriorly relative to the right ventricle in body coordinates, explaining why 'lateral' coronary veins are actually located posteriorly.

## 2 Embryological foundation: why variants happen

The CS doesn't appear fully formed. It earns its final configuration through a series of developmental events that begins in the fourth week of embryonic life. Understanding this embryology isn't an academic exercise; it explains the variants you'll encounter in about one in every twenty cases.

**Fig. 2:**
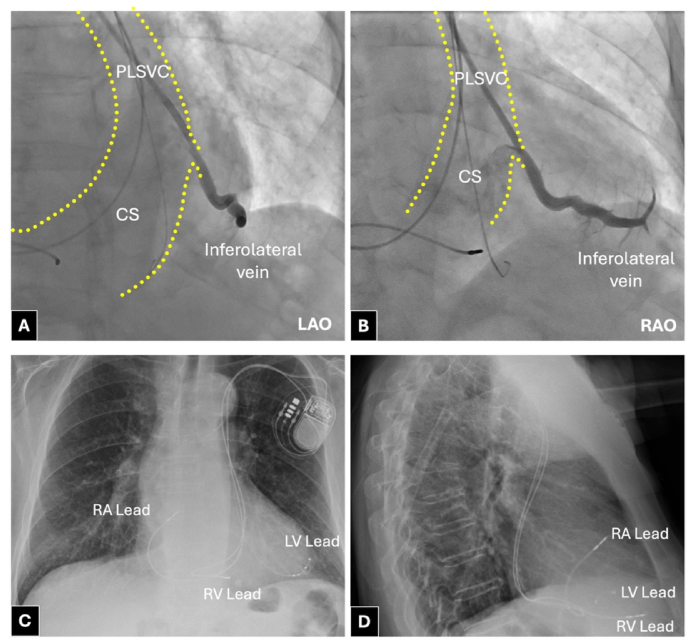
Cardiac resynchronization therapy implantation in a patient with persistent left superior vena cava (PLSVC). **(A)** Left anterior oblique (LAO) projection: selective venography of the inferolateral vein demonstrating the characteristic tortuous course of the delivery system through the dilated coronary sinus - a hallmark of PLSVC anatomy. **(B)** Right anterior oblique (RAO) projection: selective venography confirming the inferolateral trajectory along the lateral left ventricular wall. **(C)** Posteroanterior chest radiograph following CRT-P implantation. The left ventricular lead follows the characteristic "double curve" pathway: descending via the PLSVC, coursing through the dilated coronary sinus, and terminating in the inferolateral vein. Note the significant lead redundancy within the CS - an unavoidable consequence of the elongated venous route requiring careful slack management. **(D)** Lateral chest radiograph confirming final lead positions. The looping of the LV lead within the dilated CS is clearly visible. The relatively apical lead position reflects the limited tributary options often encountered in PLSVC, where mid-ventricular segments may lack adequate vein caliber or stability for reliable lead fixation. **Abbreviations:** CS, coronary sinus; CRT-P, cardiac resynchronization therapy pacemaker; LV, left ventricular; PLSVC, persistent left superior vena cava; RA, right atrial; RV, right ventricular.

The CS originates from the left horn of the sinus venosus, while the right horn gets incorporated into the right atrium (RA) [2], [3]. As the left common cardinal vein regresses, its remnant becomes the CS, and the left anterior cardinal vein contributes to the oblique vein of the left atrium, the vein of Marshall [2], [4]. This developmental pathway explains why some patients show up with a persistent left superior vena cava (PLSVC), seen in 0.3–0.5% of the general population [5]. When you encounter PLSVC, expect a massively dilated and tortuous CS that fundamentally changes your approach to sheath delivery and backup support [5], [6]. Fig. 2 illustrates successful CRT implantation in a patient with PLSVC, demonstrating the characteristic elongated lead pathway through the dilated CS and the significant redundancy that must be managed to prevent dislodgement.

The Thebesian valve, that endocardial fold that can turn a straightforward case into a long cannulation struggle, is actually a remnant of the right valve of the sinus venosus. It's present to some degree in most patients (70–82%), but in 5–16% of cases it's sufficiently obstructive to make you wish you'd brought more catheter options [7], [8], [9]. Similarly, the Vieussens valve, located near the junction of CS and great cardiac vein (GCV), represents another developmental remnant that can prevent your delivery catheter from advancing just when you thought you were already free from obstacles. The Vieussens valve is present in 65–87% of hearts [10], [11].

**Fig. 3:**
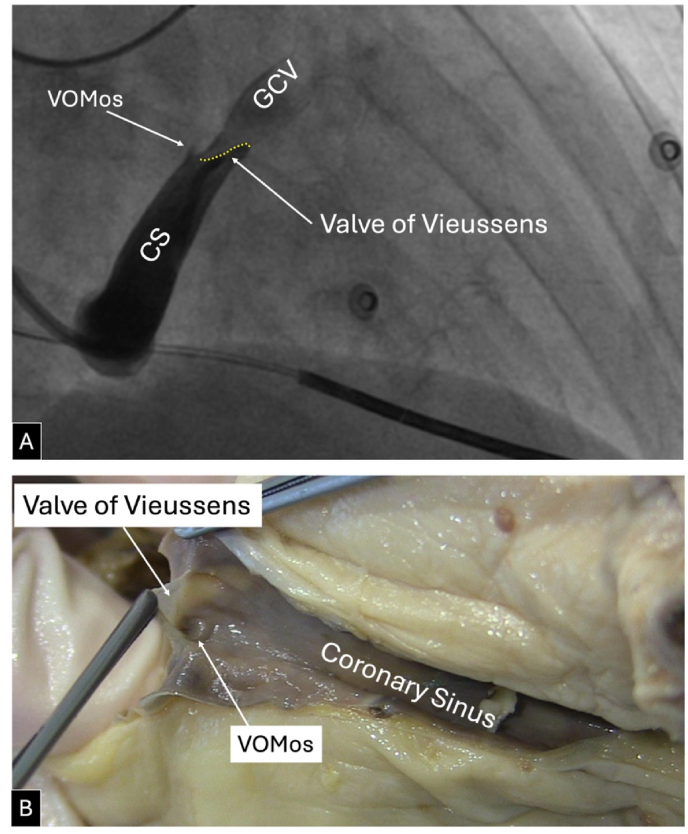
Valve of Vieussens and vein of Marshall ostium at the coronary sinus–great cardiac vein junction. **(A)** Right anterior oblique (RAO) fluoroscopy demonstrating the junction between the coronary sinus (CS) and great cardiac vein (GCV). The valve of Vieussens (dotted line) marks the anatomical boundary between these two venous segments. The vein of Marshall ostium (VOMos) is located just proximal to the valve - a consistent anatomical relationship that creates a potential hazard during catheter advancement. Forceful manipulation against a prominent Vieussens valve may inadvertently direct the catheter into the thin-walled vein of Marshall, risking perforation or dissection. **(B)** Human cadaveric specimen, diaphragmatic aspect, with the coronary sinus opened longitudinally. The valve of Vieussens is clearly visible as a membranous fold at the CS-GCV junction. The vein of Marshall ostium (VOMos) opens into the CS just proximal to the valve, confirming the fluoroscopic findings. This anatomical proximity underscores the importance of gentle technique and contrast confirmation when navigating this region.

The vein of Marshall drains into the CS just proximal to and slightly behind the Vieussens valve, a consistent anatomical relationship worth remembering [12]. This proximity creates a potential hazard during CS cannulation, particularly when using electrophysiology catheters (such as decapolar CS catheters) with blind advancement techniques. If the Vieussens valve is prominent and the Marshall vein ostium is sufficiently large, the catheter tip may inadvertently enter the vein of Marshall instead of advancing into the GCV. Given the thin-walled, fragile nature of the vein of Marshall, this misdirection can lead to perforation or dissection. Similar trauma can occur at the level of the Vieussens valve itself, where forceful catheter advancement against an obstructive valve risks intimal injury or false passage creation. These risks underscore the importance of gentle technique and contrast confirmation when navigating the CS–GCV junction, especially when resistance is encountered**.** Fig. 3 demonstrates the fluoroscopic and anatomical appearance of this critical junction.

## 3 Gross anatomy: the landmarks that matter

The CS is a thin-walled venous structure running in the inferior atrioventricular groove, typically 25–50 mm long and 6–12 mm in diameter. These numbers vary considerably based on volume status, central venous pressure, and congenital anomalies [3], [4]. Unlike other cardiac veins, the CS is covered by a myocardial sleeve composed of striated muscle continuous with both the RA and LA. This sleeve extends 25–51 mm from the ostium and in approximately 15% of cases is thickened in a sphincter-like fashion [13,14]. The CS is also unique in being bounded by valves at both ends: the Thebesian valve at the ostium and the Vieussens valve at the junction with the GCV.

A note on terminology: anatomically, the true CS is defined as the short venous segment extending from the Vieussens valve to the ostium in the RA [4]. In the electrophysiology laboratory, however, we routinely refer to the entire system including the GCV as "the CS." This convention is practical but anatomically imprecise, and worth keeping in mind when correlating procedural findings with anatomical descriptions.

The CS empties into the RA anteriorly and slightly septally to the inferior vena cava orifice. Its position at the level of the LA (not the LV) makes it our only reliable transvenous access route to the lateral LV veins and wall [4].

The CS ostium forms the inferior boundary of the triangle of Koch, but its exact orientation varies enough that you can't assume your favorite sheath will work every time. In patients with significant heart and/or RA enlargement or PLSVC, the ostium may be rotated or enlarged, fundamentally changing your approach angle and backup support [15]. More details on CS ostium localization and cannulation strategies are provided in the Catheter Techniques section below. The Thebesian valve guards this ostium in most patients, and in 10–15% of cases it's prominent enough to require a steerable electrophysiology catheter or creative catheter curves [7], [8].

## 4 The tributaries: your target zones

The lateral and inferolateral veins of the LV (traditionally called "posterolateral vein of LV") are your primary targets. They overlay the lateral and inferolateral LV walls where electrical delay is typically maximal in LBBB. The lateral vein is identifiable in approximately 91% of patients, while the inferior vein shows up in 70–76% of cases [16,17].

**Fig. 4:**
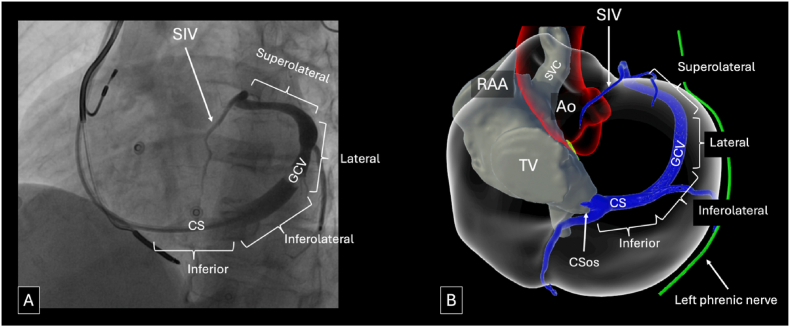
Classification of coronary sinus tributaries in the left anterior oblique (LAO) projection. **(A)** Balloon occlusion venography demonstrating the coronary sinus (CS), great cardiac vein (GCV), and the superior interventricular vein (SIV, anterior interventricular vein). Tributaries are classified by their position along the atrioventricular groove: superolateral (anterolateral), lateral, inferolateral (posterolateral), and inferior (posterior). The lateral and inferolateral veins represent the primary targets for left ventricular lead placement. **(B)** Three-dimensional computed tomography reconstruction (inHeart) in corresponding LAO orientation. Coronary veins (blue) and arteries (red) are displayed relative to cardiac chambers. The green line indicates the course of the left phrenic nerve along the lateral pericardium, and its proximity to superolateral and lateral tributaries explains the risk of phrenic nerve stimulation during cardiac resynchronization therapy. Abbreviations: Ao, aorta; CS, coronary sinus; CSos, coronary sinus ostium; RAA, right atrial appendage; SVC, superior vena cava; TV, tricuspid valve. (For interpretation of the references to colour in this figure legend, the reader is referred to the Web version of this article.)

For procedural planning and reporting, CS tributaries are classified by their position in the LAO projection (Fig. 4): inferior (draining the diaphragmatic wall), inferolateral, lateral, superolateral, and superior (the anterior interventricular vein) [18]. Lead position along the long axis of the LV is equally important and described as basal, mid-ventricular, or apical. Multiple studies have demonstrated that apical lead positions are associated with worse clinical outcomes and should be avoided when possible [19,20]. The optimal target in most LBBB patients is a basal to mid-ventricular position in a lateral or inferolateral vein, balancing electrical delay with anatomical accessibility [20].

Here's where anatomy becomes destiny: in about 15–30% of cases, tributary characteristics such as sharp takeoff angles, tortuosity, or small diameter make lead advancement difficult or impossible without inner catheters or buddy wires [16,21]. Mazur and colleagues demonstrated in their CT study that ostial diameter, angle of origin, and branch curvature independently predicted LV lead delivery success [22]. These aren't just interesting anatomical observations. They're roadmaps for your procedural planning.

The diameter of target veins ranges from 1.5 to 4.5 mm, with a median around 2.5–3.0 mm [23]. This matters tremendously when you're choosing between passive fixation, active fixation, or quadripolar leads. A mismatch between vein size and lead caliber leads to dislodgement, instability, or perforation [24]. In approximately 10–15% of patients, there is no suitable lateral vein in the target region due to unfavorable anatomy or extensive scarring, forcing you into alternative positions or advanced techniques [25,26]. However, with modern delivery systems and experienced operators, overall LV lead implantation success rates now exceed 96% [26].

Two tributaries are generally not suitable for CRT: the anterior interventricular vein (in the anterior interventricular groove) and the middle cardiac vein (MCV, in the inferior interventricular groove) [16]. The MCV, with a proximal diameter of 3–5 mm, can however serve as an alternative site for RV defibrillation lead positioning in select cases, especially in patients with mechanical tricuspid valve [27].

### 4.1 Variants you will actually encounter

Beyond PLSVC, you should be aware of CS atresia or unroofed CS—rare but absolute show-stoppers for transvenous LV pacing, sending you to the epicardial or endocardial approach [26]. Duplicate or accessory CS ostia are uncommon but clinically important when your fluoroscopy doesn't match your expected drainage pattern [4]. Other anatomical surprises include aneurysmal CS, coronary arteriovenous fistulas draining into the CS, and anomalous pulmonary venous connections, most of which show up on preprocedural CT or MRI [4].

Don't forget iatrogenic alterations: prior cardiac surgery or valve replacement can compress or redirect the CS. A mechanical tricuspid valve can displace the CS ostium or restrict your catheter maneuverability [28]. Previous epicardial leads or coronary bypass grafts create fibrosis and adhesions that fundamentally change the venous landscape.

## 5 Microanatomy: why small details cause big problems

The CS wall consists of three layers: intima, media, and adventitia. Unlike systemic veins, the media contains variable amounts of smooth muscle intermixed with elastic fibers, and in approximately 15% of individuals demonstrates sphincter-like thickening near the ostium [13,14]. Wall thickness ranges from 0.3 to 2.5 mm, with the thickest portions near the ostium and progressive thinning toward the tributaries [29]. This structural gradient has direct procedural implications: the lateral and inferolateral veins you're targeting are often extremely thin-walled, consisting of little more than a single endothelial layer over a fragile connective tissue matrix.

The venous system is low-pressure and remarkably compliant, and even when dissection occurs, it rarely leads to hemodynamic compromise [30]. In a Cleveland Clinic registry of over 5000 CRT implants, CS injury occurred in only 0.7% of cases, with perforation in just 0.12% [30]. When dissection does occur, lead implantation can still be completed successfully in about 70% of patients during the same procedure [30]. The risk increases substantially in veins smaller than 2 mm in diameter, especially when negotiating acute angles or using oversized balloons for venography.

The Vieussens valve, present in 65–87% of hearts, guards the GCV-CS junction [10], [11]. Complete obstruction is rare: only about 1–8% of valves are truly occlusive, though the valve causes catheter obstruction in up to 46% of cadaveric studies [31,32]. As discussed earlier, it can impede catheter passage if you're not expecting it, but it also serves as a useful landmark: once you're past it, you've entered the true GCV territory where target veins become accessible. In rare cases of complete obstruction, radiofrequency energy can be used to traverse this obstacle [32].

### 5.1 Subvenous epicardial adipose tissue: the hidden variable

**Fig. 5:**
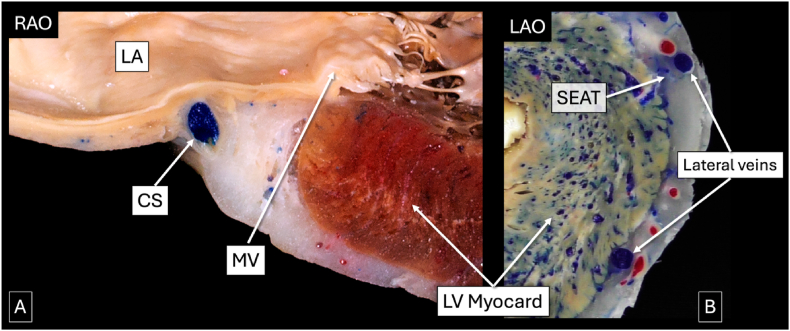
Coronary veins and subepicardial adipose tissue (SEAT): microanatomical considerations for lead placement. **(A)** Human cadaveric specimen, transverse section through the left atrioventricular groove (RAO-equivalent orientation). The coronary sinus (CS) is positioned predominantly at the left atrial (LA) level rather than midway between the atrium and ventricle, a relationship which is present in over 90% of hearts. The mitral valve (MV) annulus lies superiorly and anteriorly to the CS. **(B)** Human cadaveric specimen, transverse section through the lateral left ventricular wall (LAO-equivalent orientation). The subepicardial adipose tissue (SEAT) is visible as a layer of variable thickness between the lateral coronary veins (blue) and the underlying myocardium. Thinner SEAT in the lateral wall provides better electrode-to-myocardium contact, contributing to lower capture thresholds compared to regions with thicker adipose coverage. **Abbreviations:** LA, left atrium; LV, left ventricle; MV, mitral valve. (For interpretation of the references to colour in this figure legend, the reader is referred to the Web version of this article.)

Between the coronary veins and the underlying myocardium lies a layer of subvenous epicardial adipose tissue (SEAT) of variable thickness [33] (Fig. 5). This detail rarely appears in standard anatomy texts but has significant implications for CRT. Fat is an effective electrical insulator, and SEAT thickness directly affects both sensing amplitude and pacing capture threshold [33,34].

Morphometric studies using CT and histological analysis have quantified these differences [33]. The anterior interventricular vein has significantly thicker SEAT than lateral and inferolateral tributaries. The left marginal vein and inferolateral vein of the LV, by contrast, are underlaid by relatively thin adipose layers, typically 1–3 mm compared to 4–6 mm beneath the AIV [33].

This anatomical observation correlates with clinical experience: leads placed in lateral and inferolateral veins consistently demonstrate lower pacing thresholds and better sensing than those in anterior positions [34]. The thinner SEAT in these regions provides superior electrode-to-myocardium contact. Whether this represents a causal relationship or simply correlates with the fact that lateral veins also overlie the region of maximal electrical delay in LBBB remains debated, but the practical implication is the same. Your best target veins from an electrical standpoint are often the same ones that provide optimal resynchronization.

### 5.2 Myocardial sleeves

Myocardial sleeves extending from the LA cover the proximal CS for 25–51 mm from the ostium [13,14]. Their arrhythmogenic potential matters primarily for AF ablation. These sleeves also serve as the anatomical substrate for inferoseptal accessory pathways in Wolff-Parkinson-White syndrome: myocardial fibers extending around the CS and cardiac veins can form connections between atria and ventricles, explaining why up to one-third of inferoseptal pathways require ablation within the CS or its tributaries [35,36].

For CRT purposes, these sleeves occasionally explain unexpected electrogram morphology or elevated sensing thresholds when an LV lead sits too proximally. The extent of myocardial coverage decreases from proximal to distal CS and is virtually absent in most lateral branches [14]. If your lead demonstrates unusual signals or inconsistent sensing, consider whether it may be positioned within the sleeved portion of the CS rather than in a true tributary.

## 6 Imaging: seeing what you're getting into

### 6.1 Fluoroscopy and venography: the real-time standard

**Fig. 6:**
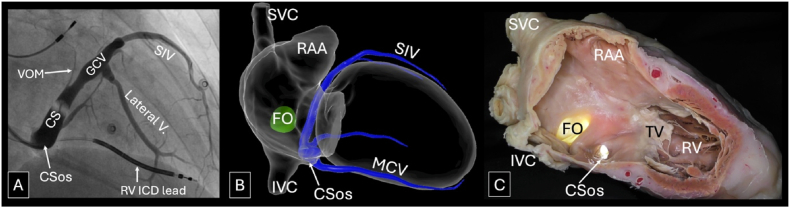
Right anterior oblique (RAO) projection: fluoroscopic-anatomical correlation for coronary sinus cannulation. **(A)** RAO fluoroscopy demonstrating coronary sinus venography with a right ventricular defibrillation lead in place. The coronary sinus (CS), great cardiac vein (GCV), superior interventricular vein (SIV, anterior interventricular vein), lateral vein, and vein of Marshall (VOM) are visualized. The CS ostium (CSos) is located inferior and anterior to the superior vena cava (SVC) and fossa ovalis (FO) in this projection. **(B)** 3D computed tomography reconstruction (inHeart) in corresponding RAO orientation. Coronary veins (blue) are displayed in relation to the right atrial structures. The fossa ovalis (FO, green circle) and CSos define key landmarks for catheter navigation. The inferior interventricular vein (middle cardiac vein, MCV) is shown draining the diaphragmatic surface. **(C)** Human cadaveric specimen, right atrium and ventricle opened, RAO-equivalent view. The spatial relationship between the CSos, FO, tricuspid valve (TV), inferior vena cava (IVC), SVC, and right atrial appendage (RAA) is demonstrated. This orientation corresponds to the fluoroscopic view during CS cannulation from a superior venous approach. (For interpretation of the references to colour in this figure legend, the reader is referred to the Web version of this article.)

Fluoroscopy remains our gold standard. LAO 30° distinguishes superior from inferior veins and assesses the lateral wall, while RAO 30° helps with septal orientation (Fig. 6). Balloon occlusion venography enhances contrast retention for visualizing lateral and inferolateral branches.

However, fluoroscopy can't reliably show vessel wall integrity, depth relative to the epicardium, or surrounding structures. Lead positions determined by fluoroscopy alone show discordance rates of 30–40% compared to CT for exact segment localization [37]. Real-time fluoroscopic guidance remains essential—you just need to recognize its limitations.

### 6.2 CT: the planning tool that changes cases

Cardiac CT provides three-dimensional visualization of vein number, trajectory, takeoff angle, and ostial diameter before you scrub in [38]. It reveals veins that venography misses and identifies anatomical barriers like Thebesian or Vieussens valve prominence [39]. Preprocedural CT reduces fluoroscopy time and improves first-attempt success rates [40].

Consider CT strongly in: prior failed CRT attempts, suspected PLSVC, dilated CS on echocardiography, congenital heart disease, and ischemic cardiomyopathy where scar location matters.

### 6.3 MRI: scar mapping over vein mapping

Cardiac MRI with late gadolinium enhancement excels at assessing myocardial viability [41]. Pacing over transmural inferolateral scar yields only 14% clinical response compared to 81% without scar [42]. MRI's value lies in identifying viable, late-activating myocardium to target and scarred segments to avoid.

### 6.4 Fusion imaging

Advanced systems enable fusion of CT or MRI with live fluoroscopy during implantation [43]. These overlays help target optimal branches, avoid scar, and confirm lead trajectory in real time—particularly valuable in complex cases and for avoiding phrenic nerve capture.

## 7 Catheter and wire techniques: navigating the anatomy

### 7.1 Getting into the CS

CS cannulation via the RA uses a guiding sheath, slittable introducer, or deflectable EP catheter, with fluoroscopic LAO and RAO guidance essential for aligning with an ostium that may be eccentrically positioned or valve-covered [44]. Sheath shape and flexibility should match RA anatomy: horizontally oriented hearts, as is typical in patients with heart failure, often require inferiorly directed curves, while vertical hearts respond better to superior curves. Steerable delivery systems accommodate these variations [45].

**Fig. 7:**
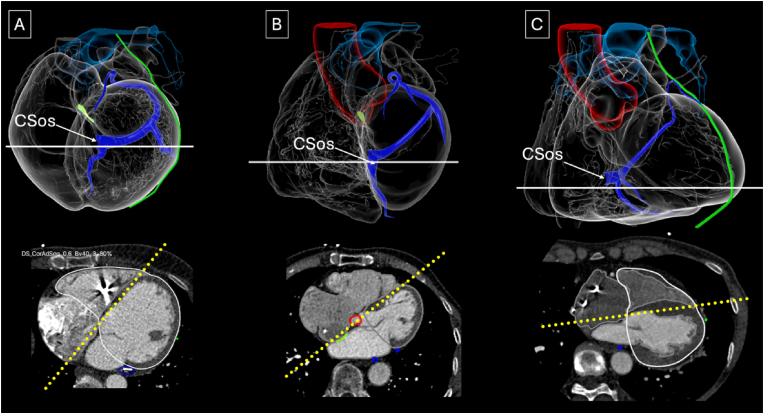
Impact of cardiac dilatation on coronary sinus ostium position: implications for fluoroscopic projections. **(A**–**C)** Upper panels: 3D CT reconstructions (inHeart) in LAO projection. The white line indicates the inferior border of the CS ostium (CSos). Lower panels: corresponding axial CT slices with yellow dotted lines indicating the cardiac long axis orientation. With progressive cardiac dilatation, two key changes occur: (1) the heart assumes a more horizontal orientation, as reflected by the increasingly horizontal cardiac long axis, and (2) the CSos displaces inferiorly relative to the cardiac silhouette in LAO. This inferior displacement explains why standard fluoroscopic angles may be insufficient in patients with dilated cardiomyopathy—the CSos appears lower within the cardiac shadow than anticipated, requiring adjustment of both C-arm angulation and catheter approach. The green line indicates the left phrenic nerve course along the lateral pericardium. (For interpretation of the references to colour in this figure legend, the reader is referred to the Web version of this article.)

**Fig. 8:**
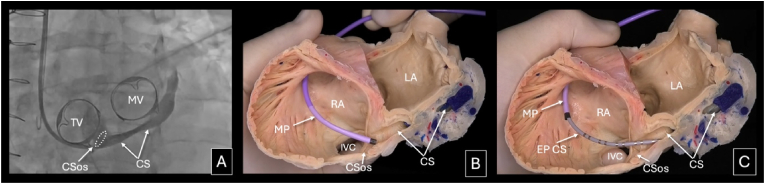
Coronary sinus cannulation techniques: fluoroscopic landmarks and catheter approaches. **(A)** Left anterior oblique (LAO) fluoroscopy in patient after mechanical tricuspid and mitral valve replacement. This image demonstrates the CS ostium (CSos) position at approximately 5 o'clock relative to the tricuspid valve (TV) annulus. The mitral valve (MV) silhouette and proximal coronary sinus (CS) are visible following contrast injection. **(B)** Human cadaveric specimen with removed ventricles and distal parts of atria at the level of CSos, LAO-equivalent view. Direct CS cannulation using a multipurpose guiding sheath alone. The CSos is located at 5 o'clock in LAO, inferiorly and anteriorly to the fossa ovalis, between the inferior vena cava (IVC) and the TV annulus. This approach is often successful when CS anatomy is favorable. **(C)** Human cadaveric specimen with removed ventricles and distal parts of atria at the level of CSos, LAO-equivalent view. Deflectable electrophysiology catheter-assisted cannulation technique. The steerable catheter is advanced through the guiding sheath to locate and engage the CSos, particularly useful when the ostium is superiorly directed or when a prominent Thebesian valve impedes direct sheath advancement. This technique also provides enhanced backup support for sheath advancement. **Abbreviations:** CS, coronary sinus; CSos, coronary sinus ostium; IVC, inferior vena cava; LA, left atrium; MV, mitral valve; RA, right atrium; TV, tricuspid valve.

In patients with dilated cardiomyopathy, the CS ostium displaces inferiorly relative to the cardiac silhouette as the heart assumes a more horizontal orientation (Fig. 7). This shift explains why standard fluoroscopic angles may be insufficient—steeper LAO projections (60–70°) are often required. A practical landmark for CS cannulation: in LAO, the CS ostium lies at approximately 5 to 5:30 o'clock in most heart failure patients with dilated hearts, shifting closer to 4 o'clock in more vertically oriented hearts. Fig. 8 illustrates both the fluoroscopic landmark and catheter-assisted cannulation techniques.

When standard approaches fail, CS diagnostic catheters provide improved torque control and safer navigation around obstructive valves or atypical ostial orientation [45]. In truly difficult cases, the "inner catheter only" technique, using a 7Fr subselector through a standard sheath, can reduce perforation risk compared to advancing large-bore outer catheters.

### 7.2 Wire selection: balancing safety and support

Hydrophilic guidewires (0.014" or 0.018") are your initial choice. Their slippery coatings facilitate advancement through tortuous anatomy, and flexibility reduces trauma risk [46]. But they lack support, limiting catheter advancement in sharply angled or reverse-turn branches.

Once you've accessed the vein, stiffer support wires (Amplatz Super Stiff, Whisper, Pilot) help deliver subselection catheters or LV leads. The buddy wire technique, using a second wire for support, improves stability and prevents system prolapse in unstable branches [46].

Branches with acute takeoff angles demand wires with angled tips or pre-shaped curves. Manual wire shaping or torque devices facilitate maneuverability through venous bifurcations or ostial ridges.

### 7.3 Subselection tools: getting where you need to go

Inner catheters or subselection catheters are critical for difficult tributaries, introduced over guidewires to provide coaxial support for lead advancement [47]. Options include the Worley Advanced LV Access System (Pressure Products), Medtronic Attain Select II, Abbott CPS Aim Universal II, Boston Scientific Acuity system, and Biotronik Selectra catheters. Various tip shapes (straight, S-curve, hockey-stick, acute, obtuse) match different anatomies, though trial-and-error remains common. Three-dimensional CT overlay techniques now help predict catheter-vein compatibility before you start.

Some subselection catheters have contrast injection ports for selective venography of individual veins when balloon occlusion venography proves incomplete. This becomes particularly valuable when you need to characterize a single target vein in detail.

### 7.4 Lead selection and delivery

Quadripolar leads have become the standard of care in CRT [48]. They offer multiple pacing vectors (10–17 configurations depending on manufacturer and device), improved success in difficult anatomy, and the ability to program around problems without reoperation. Compared to bipolar leads, quadripolar technology reduces phrenic nerve stimulation from 14% to 5%, decreases surgical revision rates, and shortens both procedure and fluoroscopy time [49].

Available quadripolar leads include the Abbott Quartet (up to 14 vectors), Boston Scientific Acuity X4 (17 vectors with compatible devices), Medtronic Attain Performa, and Biotronik Sentus. Lead selection should match the target vein anatomy. Various hook-like or spiral shapes are available in different lengths. Stabilization within the vein is accomplished by advancing the tip into a distal wedge position, then withdrawing the guidewire to release the pre-shaped configuration [48]. This increases lead adherence to the venous wall.

For large, straight vein branches or redo procedures after lead dislodgement where passive fixation is unlikely to succeed, active fixation leads with side helix deployment allow anchoring without excessive forward pressure [50]. The Medtronic Attain Stability Quad is currently the only commercially available quadripolar active fixation LV lead, demonstrating dislodgement rates of 0.7% compared to 4.7% with passive fixation leads [50]. These are particularly useful when you're working with a vein that has already failed to hold a passive lead.

### 7.5 Managing difficult anatomy

Sharp angulation and excessive tortuosity demand wire reshaping, torqueable subselection catheters, and frequent contrast re-evaluation. Minimize prolonged manipulation in thin-walled branches to reduce perforation or dissection risk.

When a vein is stenotic or won't accommodate the lead, balloon venoplasty using low-profile coronary balloons (2.0–3.0 mm) becomes an option [51]. This carries risk and is generally reserved for high-value targets in patients with limited alternatives. Start with undersized balloons and low pressures. Most operators consider venoplasty only after confirming that the target vein overlies viable myocardium and represents the best available option.

**Fig. 9:**
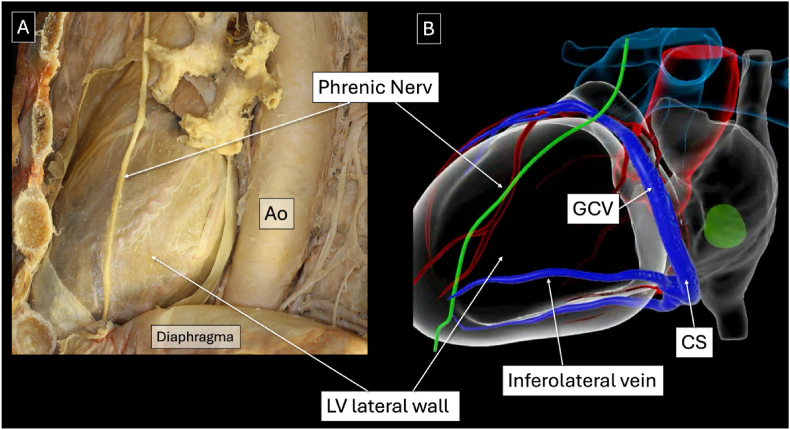
Left phrenic nerve anatomy and risk of phrenic nerve stimulation during CRT. **(A)** Human cadaveric specimen, left lateral view with pericardium opened. The left phrenic nerve courses along the lateral pericardium, descending over the left ventricular (LV) lateral wall toward the diaphragm. The aorta (Ao) is visible posteriorly. This anatomical relationship explains why pacing from lateral and superolateral tributaries carries a risk of phrenic nerve stimulation. **(B)** 3D CT reconstruction (inHeart) demonstrating the spatial relationship between the left phrenic nerve (green line) and coronary venous tributaries. The great cardiac vein (GCV) and inferolateral vein course in close proximity to the phrenic nerve path. The coronary sinus (CS) is shown at the base. Note that inferolateral veins typically offer a safer distance from the phrenic nerve compared to lateral or superolateral positions. (For interpretation of the references to colour in this figure legend, the reader is referred to the Web version of this article.)

Always test for phrenic nerve stimulation at high pacing output (10V) during implantation. The left phrenic nerve courses along the lateral pericardium, placing it in close proximity to lateral and anterolateral coronary vein branches (Fig. 9). If stimulation is present, the advantage of quadripolar leads becomes immediately apparent: reprogram to a different vector before accepting the position. If phrenic capture persists across all vectors, retract or rotate the lead into a new position. This is far preferable to discovering the problem at first follow-up.

### 7.6 Collateral veins: the retrograde option

When antegrade access to the target vein fails due to acute angulation, stenosis, or valve obstruction, vein-to-vein collaterals offer an alternative pathway. These interconnections between coronary venous branches, though often not visible on initial venography, can be identified with selective injection or blind wire exploration [52].

The technique involves advancing a hydrophilic wire through collaterals from an accessible vein back into the CS body, then snaring the wire to create a veno-venous loop. This provides stable rail support for retrograde lead advancement into otherwise unreachable targets [53]. In experienced hands, this approach can salvage cases that would otherwise require surgical epicardial leads. The key is recognizing when collaterals exist and having the interventional skills to exploit them.

## 8 When transvenous fails: alternatives

In approximately 5–10% of patients, transvenous LV lead placement is not possible due to CS anatomy, lack of suitable tributaries, or persistent phrenic nerve stimulation across all vectors [54]. Modern alternatives include conduction system pacing (CSP): Left bundle branch area pacing (LBBAP) or His bundle pacing can achieve physiological resynchronization without CS access. The 2025 ESC/EHRA consensus supports CSP as a reasonable alternative in failed transvenous CRT [55].

Surgical epicardial leads: Via limited thoracotomy or thoracoscopic approach, particularly useful in patients undergoing concomitant cardiac surgery. Hybrid approaches: LOT-CRT (LBB-optimized CRT) or HOT-CRT (His-optimized CRT) combine CSP with conventional RV pacing for optimized resynchronization [55]. The key message: a failed transvenous attempt is not a failed CRT. Multiple pathways exist to achieve resynchronization, and the implanting physician should be familiar with alternatives or prepared to refer to centers with CSP expertise.

## 9 Electrophysiological considerations: where anatomy meets electricity

### 9.1 Electrical delay parameters: Q-LV and S-QRS

The Q-LV interval—time from surface QRS onset to the local sensed electrogram at the LV lead—serves as the primary surrogate for electrical delay at the pacing site. A Q-LV greater than 95 ms consistently predicts higher CRT response, particularly in patients with LBBB [56]. This interval directly reflects anatomical lead placement: veins overlying lateral and inferolateral segments coincide with latest electrical activation and are therefore optimal targets. A coordinate system grading the mitral ring from 0° (CS ostium) to 360° has been proposed to standardize lead position reporting independent of individual CS branch morphology. During right ventricular pacing, electrical delays become predictable, with maximum delays (∼78% of QRS duration) consistently found at approximately 162° on the mitral ring—corresponding to the posterolateral (inferolateral) region at the basal segment. This provides an anatomical rationale for targeting lateral and inferolateral veins [57]. Anterior or apical positions typically show shorter Q-LV intervals and inferior outcomes [58].

The stimulus-to-QRS interval (S-QRS) provides complementary information [59]. A prolonged S-QRS (≥37 ms) indicates local conduction delay, often due to underlying scar. A site with excellent Q-LV but prolonged S-QRS may be less effective than expected—both parameters together provide a more complete picture of local tissue viability.

### 9.2 Avoiding electrical dead zones

Anatomical accessibility doesn't guarantee electrical viability. Transmural scar regions may not conduct effectively, resulting in high capture thresholds or CRT non-response [60]. Pacing over transmural inferolateral scar yields only 14% clinical response compared to 81% in patients without scar in this region [42]. Cardiac MRI with late gadolinium enhancement enables scar localization to avoid these segments.

### 9.3 Phrenic nerve capture: anatomy determines risk

Phrenic nerve stimulation occurs in up to 20% of CRT cases, usually from anatomical proximity between lateral veins and the left phrenic nerve coursing along the lateral pericardium (Fig. 9) [61]. High-output pacing (10V) at implantation detects phrenic capture. Quadripolar technology has reduced clinically significant phrenic stimulation from 14% to 5%, with most cases resolved through vector reprogramming rather than reoperation [49].

## 10 Fixation, validation, and capture: making it stick

### 10.1 Fixation strategies matched to anatomy

Most CS leads rely on passive fixation—wedging within small-diameter or tortuous branches. Successful passive fixation requires adequate distal vein length (over 30 mm), gentle curvature supporting lead tension without slippage, and absence of significant vein dilation [20].

Where passive fixation proves suboptimal—large, straight, or mobile veins—active fixation leads provide alternatives. These leads use small side helices engaging the vein wall without full penetration [34]. They provide rotational control and minimize wedging requirements, allowing placement even in otherwise unsuitable veins.

### 10.2 Position validation: beyond fluoroscopy

Anatomical positioning traditionally relies on fluoroscopy using LAO and RAO views to assess location along the LV free wall. Lateral or inferolateral positions target latest activation regions. But radiographic criteria alone can mislead.

The Q-LV interval serves as the most reliable electrical positioning indicator. Q-LV exceeding 95 ms—measured from surface QRS to sensed LV electrogram—predicts CRT response in multiple trials [30]. Fusion imaging (CT-fluoroscopy overlays or 3D electroanatomic maps) improves lead targeting by correlating anatomical location with viability or delay data [27].

## 11 Outcomes and complications: what the anatomy teaches us

### 11.1 Response rates and anatomical predictors

CRT response rates range from 60 to 75% depending on patient selection and lead placement [35]. Anatomically guided placement in veins overlying latest-activating viable myocardium correlates with improved outcomes [31,36]. Absence of suitable lateral veins may force suboptimal anterior or apical placement, associated with reduced response rates [31].

### 11.2 Lead dislodgement

Lead dislodgement occurs in 3–8% of implantations, more frequently in large-diameter, straight, or mobile veins [33]. Prevention strategies include deep wedging, loop formation, and active fixation leads in at-risk anatomies [34].

### 11.3 Coronary venous perforation

Though rare (<1–3%), perforation may cause tamponade. It typically occurs during wire or catheter manipulation in tortuous, thin-walled branches [33]. Prevention includes limiting manipulation in narrow branches and using soft-tipped wires. When perforation occurs, immediate pericardiocentesis and cessation of further attempts are critical.

### 11.4 Non-response: Anatomy's role

Up to 30–40% of patients may not respond to CRT [35]. Anatomical contributors include placement in early-activated zones, pacing into scar tissue, and suboptimal venous anatomy. Image-guided implantation targeting viable, late-activating myocardium can reduce non-response rates.

## 12 Conclusion: anatomy as the foundation of success

The coronary sinus and its tributaries aren't just conduits for LV lead delivery—they're the anatomical determinants of whether CRT succeeds or fails. Every variant valve, every acute-angled branch, every thin-walled lateral vein represents a decision point that separates optimal outcomes from technical compromise.

The evolution of CRT from a promising concept to guideline-recommended therapy hasn't changed the fundamental reality: you can't pace what you can't reach, and you can't maintain what you can't stabilize. Understanding coronary venous anatomy—from embryological development through microstructural detail to imaging-based procedural planning—transforms CRT from a technical procedure into a thoughtfully executed intervention where anatomy and physiology align.

The next time you face a challenging CRT case—the patient with no lateral vein, the massively dilated CS from PLSVC, the acute-angled tributary that won't accept your lead—remember that these aren't aberrations from normal anatomy. They're variations on the developmental theme that started when the left horn of the sinus venosus decided to persist instead of regress. Understanding why the anatomy looks the way it does helps you figure out how to work with it, not against it.

## Declaration of competing interest

The authors declare the following financial interests/personal relationships which may be considered as potential competing interests:

**Maxim Didenko** reports a relationship with Medtronic Cardiac Rhythm and Heart Failure Management that includes: employment.

**Guram Imnadze** reports a relationship with BIOTRONIK Inc that includes: board membership, consulting or advisory, speaking and lecture fees, and travel reimbursement. Guram Imnadze reports a relationship with Abbott that includes: board membership, consulting or advisory, speaking and lecture fees, and travel reimbursement.

**Christian Sohns** received research support and lecture fees from Medtronic, Abbott, Boston Scientific, and J&J MedTec; is a consultant for Medtronic, Boston Scientific, and J&J MedTec; has received grant support from the Else Kröner-Fresenius-Stiftung and Deutsche Herzstiftung.

**Philipp Sommer** reports a relationship with Abbott that includes: board membership. Philipp Sommer reports a relationship with Boston Scientific Corporation that includes: board membership. Philipp Sommer reports a relationship with Johnson & Johnson MedTech that includes: board membership. Philipp Sommer reports a relationship with Medtronic that includes: board membership.

If there are other authors, they declare that they have no known competing financial interests or personal relationships that could have appeared to influence the work reported in this paper.

The other authors declare **no** financial interests or personal relationships that could have appeared to influence the work reported in this paper.
